# Adverse drug reaction detection via a multihop self-attention mechanism

**DOI:** 10.1186/s12859-019-3053-5

**Published:** 2019-09-18

**Authors:** Tongxuan Zhang, Hongfei Lin, Yuqi Ren, Liang Yang, Bo Xu, Zhihao Yang, Jian Wang, Yijia Zhang

**Affiliations:** College of Computer Science and Technology, Dalian University of Technology, Dalian, China

**Keywords:** Adverse drug reactions, Multihop self-attention mechanism, Complex semantic information, Neural network

## Abstract

**Background:**

The adverse reactions that are caused by drugs are potentially life-threatening problems. Comprehensive knowledge of adverse drug reactions (ADRs) can reduce their detrimental impacts on patients. Detecting ADRs through clinical trials takes a large number of experiments and a long period of time. With the growing amount of unstructured textual data, such as biomedical literature and electronic records, detecting ADRs in the available unstructured data has important implications for ADR research. Most of the neural network-based methods typically focus on the simple semantic information of sentence sequences; however, the relationship of the two entities depends on more complex semantic information.

**Methods:**

In this paper, we propose multihop self-attention mechanism (MSAM) model that aims to learn the multi-aspect semantic information for the ADR detection task. first, the contextual information of the sentence is captured by using the bidirectional long short-term memory (Bi-LSTM) model. Then, via applying the multiple steps of an attention mechanism, multiple semantic representations of a sentence are generated. Each attention step obtains a different attention distribution focusing on the different segments of the sentence. Meanwhile, our model locates and enhances various keywords from the multiple representations of a sentence.

**Results:**

Our model was evaluated by using two ADR corpora. It is shown that the method has a stable generalization ability. Via extensive experiments, our model achieved F-measure of 0.853, 0.799 and 0.851 for ADR detection for TwiMed-PubMed, TwiMed-Twitter, and ADE, respectively. The experimental results showed that our model significantly outperforms other compared models for ADR detection.

**Conclusions:**

In this paper, we propose a modification of multihop self-attention mechanism (MSAM) model for an ADR detection task. The proposed method significantly improved the learning of the complex semantic information of sentences.

## Background

With the rapid growth of the number of drug types, it is essential to determine the safety of the drugs that are used. Adverse drug reaction (ADR) is a broad term encompassing the dangerous effects that a drug may have. ADRs may occur after short-term or long-term administration, or they may be produced by a combination of two or more drugs. In a study that was concluded in 2000, it was reported that approximately 7000 deaths [1] were caused by ADRs each year. The systematic review of a prospective observational study stated that 5.3% of all hospital admissions are associated with ADRs [2]. Thorough knowledge of ADRs can effectively prevent their occurrence in patients [3, 4]. Therefore, ADR detection is crucial for pharmacovigilance. Data that have been previously used in ADR research came from the Federal Drug Administration’s Adverse Event Reporting System (FAERS) [5, 6] and clinical electronic medical records. Because of the privacy protection, those kinds of databases are not fully open access. Moreover, those databases are updated slowly, which limits the prevention of adverse drug reactions.

**Fig. 1:**
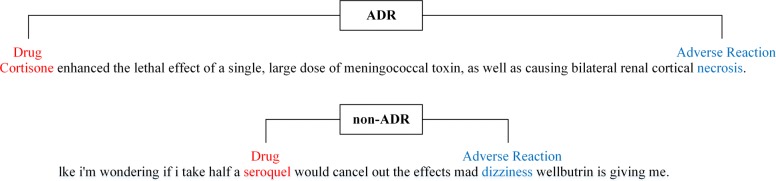
The examples of annotated sentences in the ADR corpus

Currently, due to the exponentially growing biomedical literature and the rapid development of social media, the resources that are generated are unlimited. Due to its fascinating characteristics and great potential, automatically extracting entities and their relations from the biomedical text have attracted much research attention [7]. Our research is entirely focused on biomedical text [8] and twitter messages [9]. As shown in Fig. 1, it is an example of annotated sentences from the ADR corpora, The first sentence contains ADR, and the second sentence does not contain ADR.

In early studies, researchers used the co-occurrence method [10] to determine the existence of ADRs. If a sentence includes both a drug and adverse reactions, it suggests that those terms are probably related. However, this method ignores the genuine semantic relations between drug and adverse reactions. Some researchers used rules-based approaches [11], but the rules are difficult to cover all situations. In recent years, researchers developed many systems for automatically extracting relations from biomedical text, such as protein-protein interactions and gene-disease relations [12, 13]. Meanwhile, some studies employed traditional machine-learning techniques in ADR detection [14, 15]. Bian et al. [16] built support vector machine (SVM) classifiers to analyze the potential adverse events. Liu et al. [17] developed a feature-based approach for the feature selection for adverse drug events (ADEs). However, biomedical relation detection based on traditional machine-learning heavily relies on feature engineering, which is a cumbersome process.

Recently, deep learning has attracted significant attention in natural language processing (NLP) due to its numerous advantages [18, 19], such as less feature engineering, better performances and strong representations of data compared to other systems [20]. The convolutional neural network (CNN) and recurrent neural network (RNN) are two widely used neural network structures in biomedical relation detection. Lee et al. [21] build several semi-supervised CNN models for ADE classification. Zeng et al. [22] proposed a piece-wise CNN (PCNN) method to automatically learn sentence-level features and select one valid instance for the relation classification. Li et al. [23] used Bi-LSTM to represent the sentence vector combining the contextual information. It was found that the CNN model could reduce the number of model parameters through local connections and parameter sharing. It could better extract local features from short sentences. The RNN model is designed to deal with long-distance sequences and is good at dealing with long-distance features. However, the contribution of each element in the sentence is the same. Meanwhile, there is no more prominent part of the sentence that determines the category of the ADR.

The segments with a stronger focus in the sentence are treated as more important, which would influence the sentence representation. Alimova et al. [24] investigated the applicability of the interactive attention network (IAN) for the identification of adverse drug reactions from user reviews. Lin et al. [25] and Ji et al. [26] introduced an attention mechanism to the PCNN-based multi-instance learning (MIL) framework to select informative sentences. Zhou et al. [27] introduced a word-level attention model to the Bi-LSTM-based MIL framework and obtain sgnificant result. By focusing on the most relevant part of the detection of adverse reactions, this method has a greater impact on the vector representation of sentences. Although previous approaches have promising results in ADR task, they are limited to a single sentence representation that provides single semantic information. In fact, multiaspect information needs to be considered when understanding a sentence, which is helpful to enhancing the ADR detection performance.

In this paper, we propose a multihop self-attention mechanism (MSAM) that is related to dynamic memory networks (DMNs) [28] to deal with these problems. The contributions of our work can be summarized as follows:

- Our model is different from the previous methods that use the single vector representation of a sentence, which cannot obtain adequate information about a sentence. Our model employs multiple vectors for the sentence representation by taking into account the previous memory results.
- By applying multiple attention mechanism, each attention step obtains different attention weights focusing on the different segments. This approach allows the MSAM to capture the different semantic information from the multiple representation of the sentence.
- Since a complete sentence contains intricate semantic information, our model applies multiple steps semantic analysis of the text to enhance the ADR classification performance. Via extensive experiments, the results show that our model achieves state-of-the-art ADR classification based on the sentence.

## Methods

**Fig. 2:**
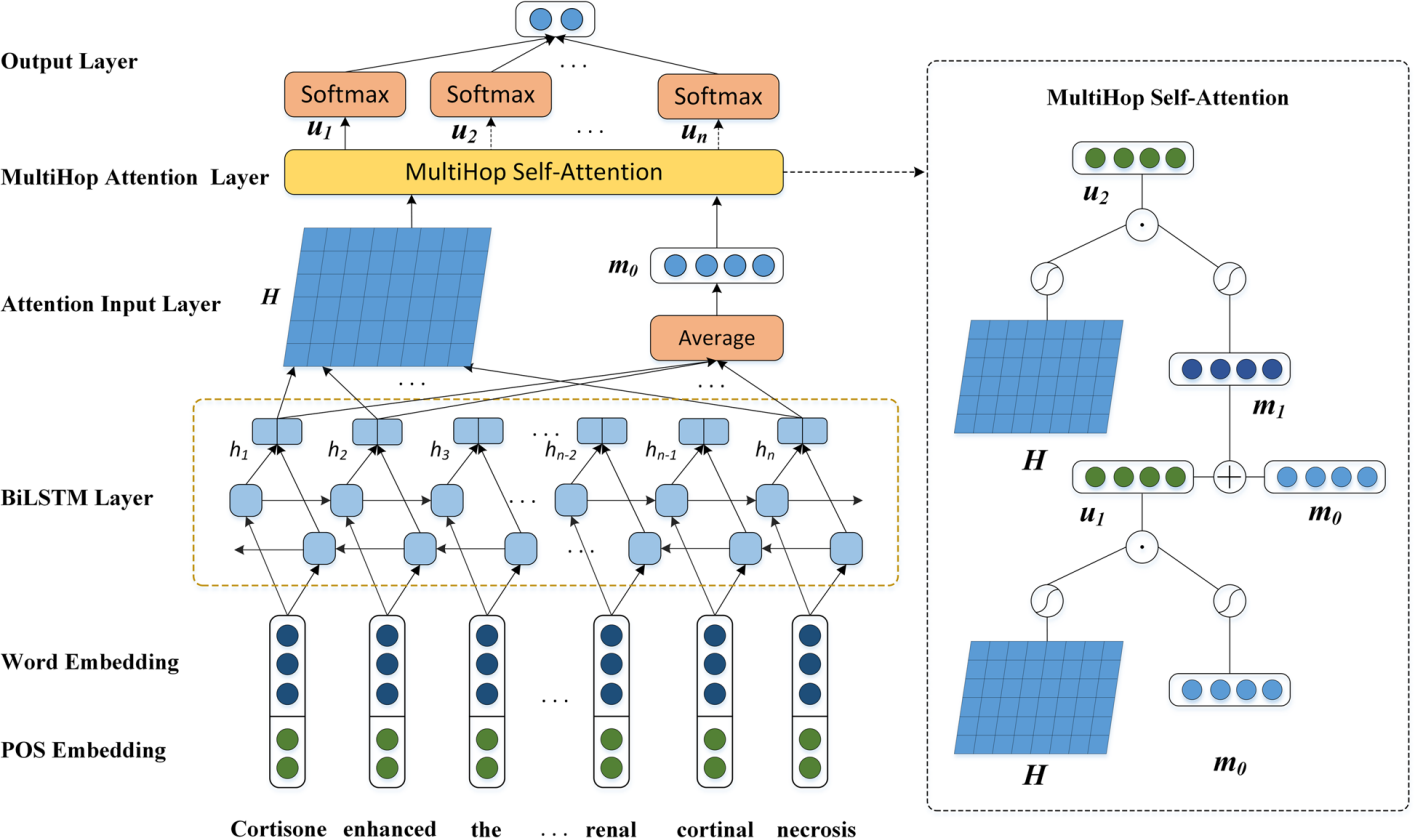
The sequential overview of our MSAM model

In this section, we explain in detail our method. First, the embedded features that are used in our neural network model are described. Second, the basic Bi-LSTM model and self-attention mechanism are introduced. At last, our MSAM model is presented. Figure 2 illustrates the MSAM that is applied to the identification of ADRs. The right side of the figure shows the details when the number of iteration steps is K =2.

The architecture of our model consists of four components: (1) The words are represented by word vector embedding and position embedding, respectively. (2) Bi-LSTM can be used for extracting the contextual information in the sentence. (3) The multihop self-attention mechanism can extract complex semantic information. (4) The output layer realizes the sentence classification.

### Embedding input representation

The input of our model is sentence sequence. Give a sentence *S=* {*w*_1_,*w*_2_,…,*w*_*n*_} denote the sentence sequence. In this paper, word *w*_*i*_ in the sentence sequence is represented by concatenating the word embedding and position embedding.

#### Word embedding

Word2Vec [29] learns low-dimensional continuous vector representations for words, which could solve the memory overflow problems that are caused by the one-hot encoding to represent the word vectors. Meanwhile, this approach could also capture the semantic information underlying the words. In recent years, word embedding has been successively applied in NLP tasks, such as sequence labeling [15], sentiment analysis [30], information retrieval [31], text classification [32] and so on. In our experiments, we downloaded a total of 2,680,617 MEDLINE abstracts from the PubMed by using the query string ’drug’. Then, these abstracts were used to train word embedding by using Word2Vec [29] as the pre-trained word embedding. The word $w^{word}_{i}$ is encoded into a real-values vector by using pre-trained word embedding.

#### Position embedding

In addition to word embedding, we also exploit position embedding to extend the input representation ability. The same word in different contexts or in different positions in a sentence has different meanings [33]. However, the word embeddings do not consider this information. Therefore, we used position embedding to capture the position features by distinguishing the relative distances between each word and the entities. For example, in the sentence “*We describe a case of EGE manifested as an allergy to gemfibrozil.*”, the relative distances from the word ’*allergy*’ to ’*EGE*’ and ’*gemfibrozil*’ are 4 and -2, respectively. Then, we mapped the relative distance to a position embedding vector. For position embedding, we randomly initialize the position vector according to a standard normal distribution and updated it when training the model. Finally, we could obtain two position embeddings $w^{pos1}_{i}$ and $w^{pos2}_{i}$, which are the position embeddings of *w*_*i*_ with respect to drug entity and adverse reaction entity, respectively. Thus, the overall word embedding representation for *w*_*i*_ is $w_{i}=\left [w^{word}_{i},w^{pos1}_{i},w^{pos2}_{i}\right ]$.

### Extract contextual information

RNNs perform well in processing sequential data benefits since the RNNs have the advantage of limited short-term memory. However, when analyzing long-distance sequences, RNNs will lose the previous information, and vanishing gradient problems will occur [34]. Long Short-Term Memory (LSTM) [35] is proposed for RNNs. It designed to deal with the long-distance sequences and solving the vanishing gradient problem.

The architecture of an LSTM unit incorporates three gates: an input gate (i), a forget gate (f), and an output gate (o). The formula of the LSTM functions are given as follows in Eqs. (1)-(6):

1 $$ f_{t}=\sigma(W_{f}\cdot[h_{t-1},w_{t}])  $$

2 $$ i_{t}=\sigma(W_{i}\cdot[h_{t-1},w_{t}])  $$

3 $$ \widetilde{C_{t}}=tanh(W_{C}\cdot[h_{t-1},w_{t}])  $$

4 $$ C_{t}=f_{t}\ast{C_{t-1}}+i_{t}\ast{\widetilde{C_{t}}}  $$

5 $$ o_{t}=\sigma(W_{o}\cdot[h_{t-1},w_{t}])  $$

6 $$ h_{t}=o_{t}\ast{tanh(C_{t})}  $$

Where *σ* and *tanh* are the activation function, and *σ* denotes the *sigmoid* function with values between 0 and 1. *W*_*f*_, *W*_*i*_, *W*_*C*_, and *W*_*o*_ are the weight matrices. *h*_*t*−1_ represents the output of the previous cell, and *w*_*t*_ represents the input of the current cell at the moment *t*. *C*_*t*_ is the memory cell at moment *t*. All of gates are set to generate the current hidden state *h*_*t*_ with the previous hidden state *h*_*t*−1_ and the input token *w*_*t*_.

The LSTM model is a sequential model. For the LSTM unit, it only learns the past information and cannot use future information. However, past or future information could impact the current word. Therefore, in this paper, forward and backward LSTM mechanisms were used to assess the valuable contextual information in the sentence. The Bi-LSTM could obtain each directional information in the sequences. The output $h_{t}=[(\overrightarrow {h_{t}};\overleftarrow {h_{t}})]$ of Bi-LSTM is a concatenation of the forward hidden state $\overrightarrow {h_{t}}$ and the backward hidden state $\overleftarrow {h_{t}}$ at time step *t*. The generated new vector *H*=(*h*_1_,*h*_2_,…,*h*_*n*_) reflects the more expressive high-level semantic meaning of the sentence. The output of the Bi-LSTM layer is a sequence of hidden vectors *H*∈*R*^*n*×2*d*^ where *n* is the sequence length and *d* is the dimensional size of the LSTM.

Language descriptions are non-standard and different. Therefore, it is especially important to find the most relevant parts of ADRs. Bi-LSTM could obtain the word dependence within the sentence and capture the internal structure of the sentence. It combines local information at a higher level through local perception. For implementation convenience, the model expects fixed-length inputs for batch processing. It is necessary to standardize the number of tokens in each sentence. In this paper, we set all sentence to be the same length by trimming longer sentences and padding shorter sentences with zero tokens. Then, we input sentence vector representation into the multihop self-attention mechanism after passing them through the Bi-LSTM layer.

### Self-attention mechanism

The importances of words in a sentence are different for the ADR detection task. However, each input word shares the same weight in the input layer of neural networks. It is necessary to assign the weight for each word according to its contribution to ADR detection. The attention mechanism was first proposed in the field of visual images [36]. Since the attention mechanism is effective in machine translation [37], many researchers have applied it to NLP. The self-attention mechanism can automatically learn the weight of each word. However, a single layer of a self-attention mechanism can only focus on one part of the sentence and ignore other key parts. Therefore, we use a multiple vectors representation that focuses on different parts of the sentence to form its overall semantic representation.

### Multihop self-attention mechanism

The first multihop attention networks were proposed in the field of machine understanding and question answering [28, 38–40]. Different parts of an answer can relate to different aspects of a question [38]. The experimental results on question answering show that multihop attention networks can achieve better performance than others. Inspired by the above ideas, our model uses multihop self-attention to improve the effectiveness of ADR tasks. The iterative nature of this multihop thinking allows it to focus on different inputs during each pass so that it can explore the intricate relationship.

In many sentences, the semantic relations between drugs and adverse reactions are various. Different parts of a sentence play different roles in ADR detection. In this section, we introduce the MSAM to predict the parameters of MSAM layers through iterations. Here, we set a memory parameter *m* and gradually update the memory parameters to iterative update the MSAM method.

Let *H*=(*h*_1_,*h*_2_,…,*h*_*n*_) denote the hidden vectors of the sequence after passing through the Bi-LSTM layer. Here, *h*_*t*_ is a concatenation of the forward hidden state $\overrightarrow {h_{t}}$ and the backward hidden state $\overleftarrow {h_{t}}$ at time step *t*. *n* is the sequence length. In each step *k*, the formulas to compute weighted representation of sentence are as follows:

7 $$ S^{k}=tanh(W^{k}_{h}H)\odot{tanh\left(W^{k}_{m}m^{k}\right)}  $$

8 $$ \beta^{k}=softmax\left(w^{k}_{S}S^{k}\right)  $$

Where *W*_*h*_, *W*_*m*_, *W*_*S*_ are the attentive weight matrices. *m*^*k*^ is a separate memory vector for guiding the next self-attention step.

The initial memory parameter vector *m* is defined based on the context vector *h*_*t*_. In each step, the sentence is represented by a vector *m*^*k*^ that specifically focuses on some aspects of a sentence. The memory parameter *m*^*k*^ is recursively updated by (9):

9 $$ \left\{ \begin{array}{lr} m^{0}=\frac{1}{N}\Sigma_{t}h_{t} \\ m^{k}=m^{k-1}+u^{k} \end{array} \right.  $$

The vector *m*^*k*−1^ is used as the input for the MSAM that is described in the previous step to extract the sentence representation *m*^*k*^. We compute the *u*^*k*^ weighted sums by multihopping the matrix *β*^*k*^ and the Bi-LSTM hidden states *H*. The resulting structured sentence representation *u*^*k*^ is shown in Eq. (10):

10 $$ u^{k}=\Sigma_{t}\beta^{k}H  $$

The sentence representation *u*^*k*^ is the weighted sum after passing through the Bi-LSTM layer hidden states *H*.

Here, we calculate the classification weight by using *u*^*k*^. Then, our model takes the average after softmax as the final classification result. The sentence probability of the ADR classification is computed as follows:

11 $$ P^{k}=softmax(ReLU(u^{k}))  $$

12 $$ P=\frac{1}{K}\Sigma_{k}P^{k}  $$

In this study, the experiments find that the best number of self-attention steps is *K*=2. In this case, each self-attention step gives a different attention distribution focusing on the different segments.

### Output and training

After we obtain the sentence representation, we predict the classification of the sample by using a fully connected network. The softmax function is chosen as the activation function, and its calculation result is between 0 and 1. The sum of these values is 1. Then, the function takes the node with the highest probability as our prediction target. The formula of the softmax function is as follows:

13 $$ S_{i}=\frac{e^{i}}{\Sigma_{j}e^{j}}  $$

Where *S*_*i*_ represents the *i*^*th*^ output value of the softmax function. Prior to the prediction, we added a full connected layer to extract key features. The cost function of the model is the cross-entropy of the true class label y defined as follows:

14 $$ C=-\Sigma_{i}y_{i}lnS_{i}  $$

Where *y*_*i*_ represents the real classification result. We trained the parameters by minimizing the loss function.

## Results

### Experimental datasets and settings

To evaluate the proposed approaches, we conduct an empirical evaluation based on two ADRs datasets: TwiMed and ADE. The two corpora have different language structures: the language in the literature is formal, but twitter language is informal with frequent misspellings and irregular grammar. Further, we briefly describe each dataset.

- **TwiMed** [9]. TwiMed corpus consists of two parts: TwiMed-PubMed and TwiMed-Twitter, which are the sentence that are extracted from PubMed and Twitters, respectively. This corpus contains three types of annotated entities: *drugs*, *symptoms* and *diseases*. In addition, it contains three types of relations between those entities: *Reason-to-use*, *Outcome-positive*, and *Outcome-negative*. In our experiments, both *symptoms* and *diseases* are considered to be adverse reactions. *Outcome-negative* is used to denote that the *drugs* in the sentence could cause *adverse reactions*. If the relationship between *adverse reactions* and *drugs* was labeled as *Outcome-negative* in the sentence, we marked the sentence as ADR (positive), otherwise, we annotate it as non-ADR (negative). The data division was similar to that used in Ilseyar et al. [24].
- **ADE** [8]. The ADE corpus is extracted from 1644 PubMed abstracts. There are 6821 sentences that contain at least one ADE (positive) and 16,695 sentences that contain no ADEs (negative), which have been divided. This corpus contains two types of annotated entities in ADE (positive) sentences: *drugs* and *diseases*. There are no annotated entities in the sentence with no ADEs (negative). Therefore, we did not annotate negative sentence in this task.

The summary statistics of corpora are presented in Table 1. As shown in this table, the ADE corpus contains significantly more annotations than TwiMed. Meanwhile, the datasets we used for the experiment included sentences in both PubMed and Twitter. Since the twitter application program interface does not allow for the sharing of actual tweet text, the published tweet data includes unique tweet ID but excludes the tweet text. Thus, it was necessary to obtain the tweet text by using web crawlers with the unique tweet ID. The original dataset contained a total of 1,000 tweets. When we reacquired the data using the IDs, only 625 (62.5%) tweets were still publicly available. The Twitter and PubMed corpora were annotated by domain experts.

**Table 1:** Summary statistics of the corpora

Coupus	Documents	ADR	non-ADR	Max sentence length	Experimental data length
TwiMed-Pubmed	1000	264	983	137	75
TwiMed-Twitter	625	311	301	64	50
ADE	1644	6821	16695	90	90

We attempt to combine different corpora to assess their classification accuracies. The annotations of the two datasets are different. First, both positive and negative data of the TwiMed corpus are annotated with entities. However, only positive data of the ADE corpus are annotated. Second, the TwiMed corpus includes twitter message data. However, the grammatical structure of twitter message is not standard, which makes it difficult to process and identify. Third, Pubmed sentences are usually longer than twitter sentences. Therefore, for the above problems, we also made corresponding adjustments in the parameter setting of our model.

In our experiments, we implemented our models using Keras and ran them on a TiTan GPU. We conducted that the average training time (seconds per sentence) of our method on the ADE, TwiMed-Pubmed, and TwiMed-Twitter corpora are 0.065 s/sent, 0.062 s/sent and 0.051 s/sent, respectively. The word embedding parameters of our model are initialized using 100-dimensional pre-trained word embeddings. The dimensionality of position embedding is 10. The model parameters are optimized using the Adam optimizer with a learning rate of 0.01. We used a maximum of 35 epochs to train the MSAM on each dataset. We set the batch sizes of the TwiMed and ADE dataset to 8 and 16, respectively. The number of hidden units for the Bi-LSTM layer is 50 when using Pubmed (and 20 for twitter). The best results are obtained when the number of self-attention steps is *K*=2.

All models were evaluated by using 10-fold cross-validation on the training set. We evaluate the performance of the classification techniques using the precision (P), recall (R) and F-score (F1), which are the major evaluation metrics for ADR detection on both corpora. The outcome F1 could quantify the overall performance by balancing the precision and recall.

### Experimental results

In our experiments, we evaluated our proposed model via the ADR detection task, which is considered to be a classification task. In previous works, most relation detection methods assess models using large corpora, and the various semantic information inside the sentences is also ignored. In contrast, our MSAM model is designed to alleviate this problem using multiple self-attention mechanism. In our experiments, the two corpora and previous methods were compared.

#### Evaluation on TwiMed

We compare our proposed model with the latest models using the TwiMed corpus. Table 2 shows the performance comparisons of various models on the TwiMed corpus.

**Table 2:** Classification results of the compared methods for the TwiMed corpus

Method	TwiMed-PubMed			TwiMed-Twitter		
	P	R	F1	P	R	F1
Feature-rich SVM [24]	0.799	0.681	0.728±0.100	0.752	0.810	0.778±0.047
IAN [24]	0.878	0.738	0.792±0.016	0.836	0.813	0.824±0.042
CNN-based method [42]	0.849	0.831	0.835±0.060	0.739	0.788	0.761±0.061
multichannel CNN [43]	0.861	0.780	0.816±0.072	0.738	0.841	0.780±0.054
Joint AB-LSTM [44]	0.817	0.856	0.831±0.040	0.701	0.828	0.754±0.072
BiLSTM+MSAM+position	0.858	0.852	0.853±0.057	0.748	0.856	0.799±0.046

In the first two lines of Table 2, we assess the performance of the main model and baseline that was proposed by Alimova et al. [24] The feature-rich SVM method is based on the SVM with a linear kernel [41]. It considered a set of features. However, in our method, we can still get better results with a few features. In the second line, the method utilized an interactive attention network (IAN) [24] to learn the representations for targets and contexts. The IAN used attention mechanisms to detect the important words of the target expression and its full context. In addition, we consider the relative position between each word in the sentence and the entity.

From the third to the fifth lines of Table 2, the methods are our implementations. Liu et al. [42] and Quan et al. [43] proposed CNN-based methods for the relationship detection task. Kumar et al. [44] presented one model, the Joint AB-LSTM based on the LSTM network. The models merge the semantic meanings to one single vector. However, our model uses multihop ideas to focus on the different segments of a sentence and obtain complex semantic information.

In the last line of Table 2, we give the experimental result of our proposed MSAM model. The results show that MSAM performs better than the baseline model in ADR classification. Compared with the IAN, our method obtains a 6.1% better F1 score on the TwiMed-PubMed corpus. Compared with Liu et al.’s [42] method, our method provides a 1.8% better F1 score on the TwiMed-PubMed corpus. However, the performance on TwiMed-Twitter is less pronounced. The reason is that the format of tweets is different from that of biomedical text, and a small amount of twitter data from only 625 tweets were still publicly available.

These experimental results suggest that our MSAM model could combine the contextual features that are extracted by Bi-LSTM. Compared with the feature-rich SVM [24] method, our method effectively reduces feature construction. We apply multiple self-attention steps to learn the representations for sentences. It can extract different important information in the sentence through each iteration. The multiple vectors that focus on different parts of the sentences could better represent the overall semantics. Therefore, the MSAM is better at capturing the complex semantic relations between drugs and adverse reactions and improving the results of the experiment.

#### Evaluation on ADE

We also compare our proposed model by using another corpus. Table 3 shows the performance comparisons of various models on the ADE corpus.

**Table 3:** Classification results of the compared methods for the ADE corpus

Method	P	R	F1
Knowledge-based system [45]	0.421	0.763	0.543
Feature-rich classification [46]	-	-	0.812
Bi-LSTM-RNN [23]	0.675	0.758	0.714
CNNA [47]	0.815	0.838	0.826
C-LSTM-CNN [48]	0.816	0.834	0.824±0.009
BiLSTM+MSAM	0.847	0.855	0.851±0.013

In the first five lines of Table 3, we present the performance of the main model and the baselines for the ADE corpus. Kang et al. [45] developed a knowledge-based relation detection system that could be successfully used to extract adverse drug events from biomedical text. The learning process relies on external knowledge and ignores sentence-specific information because of the utilization of a small amount of data. Due to the limitations of the manual rule setting, this method resulted in a high recall score but a low precision score. Sarker et al. [46] relied on generating a large set of features representing the semantic properties from the text. However, our method only used the word embedding feature on the ADE corpus, and it could still obtain better performance. Li et al. [23] investigated joint models for simultaneously extracting drugs, diseases, and adverse drug events. It used a dependency parser, which we did not need. Huynh et al. [47] proposed the convolutional neural network with attention (CNNA) by adding the attention weights into convolutional neural networks. Song et al. [48] presented the Context-LSTM-CNN method for sentence classification. The method analyzed the data based on the abstract text that contained the data. The generalization of the method is reduced, which could not be well applied to the processing of short text data, such as twitter messages.

In the last line of Table 3, we give the experimental results of our proposed MSAM model. The memory parameter *m* can record the important information of each iteration step of the multihop attention mechanism so that we can obtain multiple sources of information and comprehensively judge it. From the results, we observe that the MSAM model achieve the best results on the ADE corpus. Our results were better than others with a few features. Compared with the Context-LSTM-CNN method that achieves state-of-the-art results, our method obtains a 2.7% better F1 score.

#### Performance with different attention

In Table 4, we give experimental results of the different attention [49] models on TwiMed and ADE, respectively.

**Table 4:** Performances obtained by using different attention mechanisms

Method	TwiMed-PubMed			TwiMed-Twitter			ADE		
	P	R	F1	P	R	F1	P	R	F1
Self-Attention	0.855	0.845	0.846	0.731	0.793	0.751	0.845	0.848	0.847
Multi-head Self-Attention	0.829	0.850	0.841	0.767	0.800	0.784	0.820	0.851	0.836
Multihop Self-Attention	0.858	0.852	0.853	0.748	0.856	0.799	0.847	0.855	0.851

We can see from Table 4 that the results of the model obtained using multihop self-attention are better than those obtained by models using multi-head self-attention and self-attention. Our method allows the model to assess the information from different positions. The vector representation for each step in our model takes into account the results of the previous step. Our MSAM model can learn a better sentence representation by focusing on different aspects of the sentence, which makes the sentence-level multihop self-attention mechanism have a better chance of selecting the sentences containing ADRs. Therefore, the results of our model will be relatively better than those of others.

#### Effect of various modules

In Table 5, we also give experimental results of the basic model on TwiMed and ADE. The simplified models are described as follows:

- **Bi-LSTM**: The model is used as the baseline model. Others methods are based on this method. Forward and backward LSTM mechanisms extract the information in the sentence.

**Table 5:** Performance of various modules on the TwiMed corpus

Method	TwiMed-PubMed			TwiMed-Twitter		
	P	R	F1	P	R	F1
BiLSTM	0.853	0.806	0.829	0.680	0.754	0.715
BiLSTM+position	0.843	0.825	0.836	0.809	0.654	0.723
BiLSTM+Self-Attention+position	0.855	0.845	0.846	0.731	0.793	0.751
BiLSTM+MSAM+position	0.858	0.852	0.853	0.748	0.856	0.799
- **Bi-LSTM+Self-Attention**: The model integrates the self-attention mechanism based on the Bi-LSTM model.
- **Bi-LSTM+Multihop Self-Attention**: The model integrates the multihop self-attention mechanism based on the Bi-LSTM model. We conducts experiments for different iteration steps.
- **Bi-LSTM+Multihop Self-Attention+position**: The model integrates the position feature based on the Bi-LSTM+Multihop Self-Attention model.

Table 5 shows the results when we evaluate the position feature in experiments on the TwiMed corpus. Considering the position feature on the TwiMed corpus, the contextual information of each word in the sentence can be distinguished. The precision and recall of TwiMed-Twitter fluctuate quite widely bacause of the small amount of twitter data, further, social media language is highly informal, and user-expressed medical concepts are often nontechnical.

Table 6 shows the results for the ADE corpus when there is no position feature. The reason for these results is that the negative data in ADE corpus are not annotated. Therefore, we do not consider that a sentence may contain different relationships in the ADE corpus. Our method achieved a high F1 score of 85.1% F1, which is 0.4% better than that of the Bi-LSTM+Self-Attention method. The self-attention results are also very high because the ADE corpus contains simple information. Therefore, the results of our MSAM model on the ADE corpus are not substantially different from the results of the self-attention mechanism.

**Table 6:** Performance of various modules on the ADE corpus

Method	P	R	F1
BiLSTM	0.812	0.822	0.817
BiLSTM+Self-Attention	0.847	0.848	0.847
BiLSTM+MSAM	0.847	0.855	0.851

#### Effect of the number of MSAM steps

Table 7 shows the F1-measure with respect of the number of steps on the TwiMed corpus and ADE corpus. Step1, step2, and step3 represent the MSAM iteration steps. When the number of MSAM iteration steps is *K*=2, the model obtains the best performance. This effect might be due to the sentences not being particularly long and often containing two important aspects at most. Therefore, multiple steps may have significant effects on the multi-aspect information detection for long text. Table 7 also demonstrates that the performances on the different ADR corpora varied significantly with respect to the different numbers of steps.

**Table 7:** Effects of different number of steps and self-attention on both corpus (F1)

Method	TwiMed-PubMed	TwiMed-Twitter	ADE
step1	0.831	0.786	0.819
step2	0.853	0.799	0.851
step3	0.820	0.789	0.820

#### Effect of imbalance data

We also get the result from up-sampling and down-sampling that the ratio of positive and negative samples is 1:1. Table 8 shows the performance on the TwiMed-PubMed and ADE corpora. The TwiMed-Twitter corpus does not have imbalance data, so we did not apply up-sampling or down-sampling to it. The up-sampling method copies the positive samples in the training dataset. Because of the small number of examples, increasing the positive examples could improve the experimental results to some extent. The down-sampling method removes the negative examples. Although some negative examples have been removed, which reduced the impact of noisy data, the small amount of data is the main reason why the results are not ideal.

**Table 8:** Effects of up-sampling and down-sampling for imbalanced data

Corpus	P	R	F1
TwiMed-PubMed	0.858	0.852	0.853±0.057
TwiMed-PubMed (up)	0.851	0.889	0.867±0.032
TwiMed-PubMed (down)	0.862	0.842	0.849±0.033
ADE	0.847	0.855	0.851±0.013
ADE (up)	0.846	0.869	0.857±0.007
ADE (down)	0.823	0.862	0.842±0.014

#### Case study

**Fig. 3:**
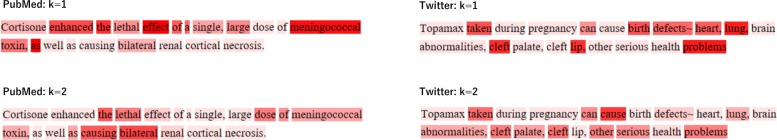
Attention heat map from MSAM (*k*=2) for ADRs classification

Figure 3 depicts the heat map of a sentence from the TwiMed corpus that was subjected to MSAM. We gave examples from PubMed and Twitter that illustrate the effectiveness of our proposed model. The stronger the red color of a word in the sentence is, the larger the multihop self-attention layer weight of that word. The first sentence is the heat map result that was obtained by MSAM step 1. The second sentence is the heat map result that was obtained by MSAM step 2. In this example, we observe that our model is able to extract the complex semantic information from the sentence. We do not only focus on entities, which are drugs and reactions, but we also focus on finding words other than entities that can play important roles in the classification of relationships. The focus of each iteration step is different, which allows the sentence vector representation to be obtained from the multidimensional perspective.

## Conclusion

Most of the neural network models only capture simple semantic information from the single representation of a sentence, which limits the performance of the ADR detection task. In fact, determining the relationship between drugs and adverse reactions requires complex semantic information. In this paper, we propose a multihop self-attention mechanism for the ADR detection task, which allows the model to capture multiple semantic information bits for the ADR detection task. By using the multistep attention mechanism, our model learns multiple vector representations that focus on different semantic information to detect the relationships between drugs and adverse reactions. Experimental results obtained for two different widely used corpora demonstrate that (i) our MSAM is effective at capturing the complex semantic information in a sentence; and (ii) our model is robust and suitable for different types of text. It is encouraging to see that our model achieves state-of-the-art results on ADR classification based on the sentence level.

Although our model achieved the best performance on the TwiMed-PubMed and ADE corpora, there is still room to improve. The performance on the TwiMed-Twitter corpus is relatively lower than that on TwiMed-PubMed. The reason for this discrepancy is that the number of training examples in the TwiMed-Twitter corpus is very limited compared with the TwiMed-PubMed corpus. Because of the particularity of twitter data, we have less available data. We obtain 625 sentences for the experiment. Meanwhile, the language in social media is highly informal. In future work, we will combine twitter data and biomedical literature data to train the model to solve the problem of insufficient twitter data.

## Data Availability

The datasets analysed during the current study are available in the https://www.ncbi.nlm.nih.gov/pmc/articles/PMC5438461/bin/jmir_v3i2e24_app1.ziphttps://sites.google.com/site/adecorpus/home/document.

## Abbreviations

ADEs: Adverse drug events
ADRs: Adverse drug reactions
Bi-LSTM: Bidirectional long short-term memory
CNN: Convolutional neural network
DMNs: Dynamic memory networks
FAERS: The federal drug administration’s adverse event reporting system
LSTM: Long short-term memory
MSAM: Multihop self-attention mechanism
NLP: Natural language processing
PCNN: Piece-wise convolutional neural network
RNN: Recurrent neural network
SVM: Support vector machine
